# Comparison of Clinical and Radiographic Success between MTA and Biodentine in Pulpotomy of Primary Mandibular Second Molars with Irreversible Pulpitis: A Randomized Double-Blind Clinical Trial

**DOI:** 10.1155/2022/6963944

**Published:** 2022-07-12

**Authors:** Alireza Eshghi, Maryam Hajiahmadi, Mohammad Hossein Nikbakht, Mona Esmaeili

**Affiliations:** ^1^Department of Pediatric Dentistry, Dental Research Center, School of Dentistry, Isfahan University of Medical Sciences, Isfahan, Iran; ^2^Student Research Committee, School of Dentistry, Isfahan University of Medical Sciences, Isfahan, Iran

## Abstract

**Introduction:**

Among the new therapeutic materials, MTA and Biodentine are recommended for pulpotomy and sealing the pulp. Considering the similar characteristics of these two materials and considering that their effects on the treatment of primary second molars with irreversible pulpitis have not been compared properly, this study aimed to compare clinical and radiographic success between MTA and Biodentine in pulpotomy of primary mandibular second molars with irreversible pulpitis.

**Materials and Methods:**

This study was conducted as a randomized double-blind clinical trial. Participants were selected according to inclusion criteria and 52 samples were randomly selected using random numbers table in group A. Then, patients in the next group B were matched with the first group in terms of age range and sex. In group A, the remaining pulp was covered with 2 mm MTA+ and in group B with 3 mm Biodentine. Participants were called for clinical evaluation every three months for 12 months (long-term follow-up). Radiographic evaluations were in the sixth and twelfth months.

**Results:**

Fischer's exact test showed that there was no significant difference between MTA and Biodentine in terms of clinical and radiographic success rates (*P* value = 1). According to the results of the Kaplan–Meier test, the survival rate in both pulp treatment methods was similar in symptomatic teeth.

**Conclusions:**

The results of this study showed that Biodentine properties are similar to MTA, and both materials show high clinical and radiographic success rates in long-term follow-up.

## 1. Introduction

American Association of Endodontists (AAE) Consensus Conference Recommended Diagnostic Terminology defines irreversible pulpitis as a clinical diagnosis based on subjective and objective findings indicating that the vital inflamed pulp is incapable of healing. Additional descriptors include lingering thermal pain, spontaneous pain, referred pain, or no clinical symptoms but inflammation produced by caries, caries excavation, and trauma” [1]. Pulpitis can be managed with different treatment options such as direct pulp capping, pulpotomy, or root canal treatment [2]. Several variables such as medical history and age, whether it is a permanent or primary tooth, exposure of the pulp, contamination with saliva, and previous restorations affect treatment decisions [3].

Root canal treatment has an excellent prognosis and is the most common emergency treatment protocol for pain relief or irreversible pulpitis in permanent teeth [4]. However, it is important to maintain the pulp of primary teeth until their physiological resorption [5]. Pulpotomy is a common procedure for treating cariously exposed pulps in primary molar teeth [6], and its positive outcomes are well documented [7]. It can be defined as the removal of the coronal pulp and placement of a therapeutic material to maintain the health of the remaining tissue [8]. The reason for using pulpotomy is the removal of that part of the coronal pulp, which is contaminated with microorganisms, due to the carious exposure and inflammation and degenerative changes seen in it. Removal of this contaminated area allows the healing process to be performed at the entrance of the pulp canal, containing the normal pulp [7].

The ideal therapeutic material should be harmless for cells and oral structures, bactericidal, promote healing of the pulp tissue, and not interfere with physiologic root resorption [6, 9, 10].

Mineral trioxide aggregate (MTA) is a widely used material in vital pulp therapy because of its ability to maintain pulp vitality and its ability to induce hard-tissue formation in pulpotomy treatment [5, 11]. It can produce effective dentinal bridging in a short time period with less pulpal necrosis and inflammation [12, 13]. MTA presents high clinical, radiographic and histological success [6, 10, 14] but it has a considerable cost. In addition, MTA has a long setting time, difficult handling, and discoloration. Pulpotomy is considered a low-cost technique that conflicts with MTA's high price [15], so researchers are trying to find a low-cost pulp capping material with success rates similar to MTA [16].

Biodentine (BD) is a material developed with active bio-silicate technology. Its powder includes zirconium oxide, tricalcium silicate, and calcium carbonate, and its liquid is mostly water with calcium chloride and a water-soluble polymer [17]. Previous studies showed the positive effects of BD on vital pulp cells, for early formation of reparative dentin and stimulating tertiary dentin formation [18, 19]. BD exhibits excellent biological properties like MTA and can be placed near the dental pulp [20].

MTA is an ideal material for pulp treatment, with high clinical and radiographic success. On the other hand, difficult handling, long setting time, high cost (about 2 times more expensive than BD in Iran), presence of toxic elements in composition, and discoloration of the tooth are disadvantages of MTA [21]. BD has beneficial characteristics but its application in the treatment of primary mandibular second molars with irreversible pulpitis has not been compared with MTA properly. The aim of this study was to compare the clinical and radiographic success between MTA and Biodentine in pulpotomy of primary mandibular second molars with irreversible pulpitis in 3–6 years old children with 3, 6, 9, and 12 months follow-up.

## 2. Method

### 2.1. Ethical Approval

This randomized double-blind clinical trial was approved by the Research Ethics Committee of the Isfahan University of medical sciences (IR.MUI.RESEARCH.REC.1400.078) and the procedure was also registered online (Iranian Registry of Clinical Trials ID: IRCT20210419051016N1).

### 2.2. Participants

In order to conduct this study, 52 children aged 3–6 years who did not have any systemic disease were referred to the pediatric ward of Isfahan Dental School in 2020 for treatment of the pulp in primary mandibular second molars with irreversible pulpitis and were selected. The reason for choosing second primary molars in the mandible was to be able to measure treatment failure criteria, such as external root resorption and periodontal ligament dilation in radiographic follow-ups, due to the limited number of anatomical landmarks and fewer radiographic superimpositions in the mandible that help evaluation with more confidence. Children and their parents were informed about the study process orally and in writing, and informed consent was obtained.

### 2.3. Inclusion and Exclusion Criteria

The patient should not have systemic diseases so as not to create a limit for anesthesia. There should be vital primary mandibular second molars with deep crown caries that was not more than one millimeter below the gingiva. Teeth had a history of typical irreversible pulpitis pain (the patient's chief complaint was spontaneous pain that lasts for more than a few seconds) and were sensitive to cold and heat. All teeth were vital, and the practitioner checked the pulp hemorrhage and the vitality of the tooth. Only patients included in the study had a possibility of a 12-month follow-up. Radiographs were taken and teeth with the following features were included in the study: pulp opening due to caries, no internal root resorption, no external pathological root resorption, no periapical radiolucency, no periodontal ligament (PDL) widening, no furcal radiolucency, and no calcaneal degeneration of the pulp. Teeth that could not be properly restored and patients who did not attend follow-up sessions were excluded from the study. Patients were able to quit the study whenever they wanted.

### 2.4. Sample Size

The sampling method was randomized and then matched. The following formula is used to determine the sample size in each group, assuming the number of samples in each group is equal. Sample size information is taken from a study by Asgary and Eghbal [22] (*α* = 0.05).

(1) $$\begin{array}{c} N=\frac{{\left(Z_{1-\left(\alpha /2\right)}+Z_{1-\beta }\right)}^{2}\left[p1\left(1-p1\right)+p2\left(1-p2\right)\right]}{d^{2}},\\ N=\frac{{\left(1/96+0/84\right)}^{2}\left[0/5\left(1-0/5\right)+0/5\left(1-0/5\right)\right]}{0/40^{2}}=25. \end{array}$$

In this study, 58 samples were used because of the probability of patients not referring to follow-up examinations. Among the samples, two in the third month of follow-up, one in the sixth month of follow-up, and three in the twelfth month of follow-up did not participate in the study process. Finally, analyzes were performed based on data from 52 of them.

### 2.5. Randomization and Allocation Concealment

Participants were admitted according to the inclusion criteria. They were randomly selected for group A using a random number table. Then, patients in the next group B were matched with the first group in terms of age range and sex. For each group of patients, a different material was used to treat the pulp.

### 2.6. Blinding

This study was a randomized double-blind clinical trial, so patients and the dentist did not know the type of material used for each tooth. The nurse prepared the materials and provided them to the dentist. The dentist used it for patients without knowing what material he was using. The follow-up procedure and evaluation of the outcomes were performed by another dentist to prevent possible biases due to patient recognition.

### 2.7. Intervention

First, periapical radiography was obtained from the target tooth using a standard parallel technique with the help of a Rinn XCP film retainer (Dentsply, USA). Film number zero with *E* speed (Kodak, Ektaspeed) was used. First, appropriate local anesthesia was performed using 2% lidocaine and 1/80000 epinephrine (Darou Pakhsh, Tehran, Iran). Then, the tooth surface was cleaned with 0.2% chlorhexidine (Shahr Daru, Tehran, Iran) and the carious crown was removed under sterile conditions and provided with angle round no. 4 bur by providing isolation by Rabradm. In the next stage, the roof of the pulp chamber was removed with 330 high-speed burs (Tizkavan, Tehran, Iran) along with water spray and the crown access was completed. Using a bur round no. 6 (Tizkavan, Tehran, Iran), the crown pulp was completely removed from the canal entrance and the pulp chamber was washed with normal saline. Homeostasis was obtained at the site of coronal pulp amputation at the entrance to the canals using a sterile cotton ball moistened with normal saline for 5 minutes. If homeostasis did not occur, the tooth would be removed from the study.

For each group, MTA or Biodentine was placed on the floor of the pulp chamber to flood the canals and prevent bacteria from entering. In group A, the remaining pulp was covered with two millimeters of MTA + past (Cerkamed Medical Company, Poland). MTA paste was obtained by mixing the powder with sterile saline in a ratio of 3 : 1. In group B, the remaining pulp was covered with 3 mm of Biodentine paste (Septodont, Saint-Maur-des-Fosses Cedex, France). The application of the substance was that five drops of liquid were poured into the capsule and then the capsule was mixed in an amalgamator at a speed of 4000 rpm for 30 seconds. In both groups, a layer of Zonalin (temporarily Golchadent company) was temporarily placed and the patient was re-visited seven days later (short term follow-up). Swelling, looseness, and fistula were examined and in the absence of these problems, the tooth was repaired with stainless steel crowns (SSC).

A dentist performed interventions for all the patients who participated in this study.

### 2.8. Follow-Up

Children were called for clinical evaluation every three months for up to 12 months (long-term follow-up). Radiographic evaluations were in the sixth and twelfth months. It should be noted that clinical and radiographic evaluations were performed by a person who did not know the type of material used in each group and did not treat patients. Periapical imaging of all treated molars was carried out with film number zero with *E* speed (Kodak, Ektaspeed) and a parallel technique, similar to preoperative imaging.

### 2.9. Outcomes Measures

**Figure 1:**
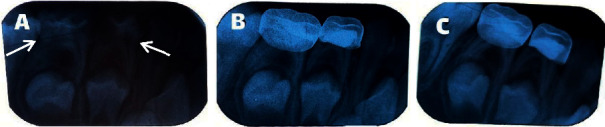
(a) Pretreatment radiography, (b) radiographic success in 6-months follow-up, (c) radiographic success in 12-months follow-up (treated teeth are marked with arrows; the second molar is intended for evaluation).

Clinical success was considered when no pain, tenderness, swelling, fistula, or pathological loosening were observed. Radiographic success was characterized by a lack of evidence of root radiolucency, internal and external resorption, bone resorption, lack of integrity of the lamina dura, and PDL widening (Figure 1).

**Figure 2:**
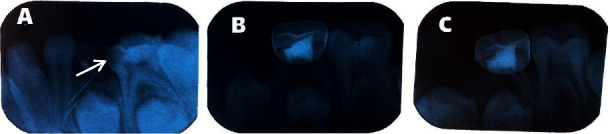
(a) Pretreatment radiography, (b) external root resorption in 6-months follow-up, and (c) external root resorption in 12-months follow-up (treated tooth is marked with an arrow).

Treatment failure was characterized by one or more of the following symptoms: internal and external root resorption, furcal radiolucency, periapical bone destruction, lack of integrity of the lamina dura, pain, swelling, and sinus tract (Figure 2).

Pulp canal obstruction was not considered a failure because it is an odontoblastic activity for dentine production [11].

### 2.10. Statistical Analysis

Data were analyzed in the SPSS software version 25. The data, clinical, and radiographic results were analyzed by the Fisher's exact test. Finally, the data were statistically analyzed by the survival test by the Kaplan–Meier method. The significance level was considered *α* = 0.05.

## 3. Results

The sample consisted of 52 children aged 3–6 years who were in two groups of 26 people. The mean age of the samples in the first group (MTA) was 5.10 ± 0.93, in the second group (Biodentine) was 5.07 ± 0.94, and in all samples was 5.08 ± 0.93.

Percussion sensitivity, PDL widening, and external root resorption were observed in some patients. None of the studied patients showed clinical and radiographic examinations, spontaneous pain, abscess, pathological loosening, periapical and furcal radiolucency, internal root resorption, and lack of integrity of the lamina dura during 12 months (Table 1).

In the sixth month of follow-up, one in the MTA group (3.85%), and in the twelfth month, one in the MTA group (3.85%), and one in the Biodentine group (3.85%), had tenderness. Fisher's exact test showed that the frequency distribution of accuracy of sensitivity between MTA and Biodentine materials in terms of time in both cases is not significant (*P* value = 1). One patient in the MTA group (3.85%) in the twelfth month of follow-up and one patient in the Biodentine group (3.85%) in the sixth month of follow-up showed dilation of the periodontal ligament. Fisher's exact test showed that the frequency distribution of periodontal ligament dilation between the two materials in terms of time in both cases is not significant (*P* value = 1). There was only one case of external root resorption in the Biodentine group (3.8%) after 12 months of follow-up. Fisher's exact test showed that there is no significant difference between the two substances in terms of the frequency distribution of external root resorption (*P* value = 1) (Table 1).

The treatment failure rate was 11.54% (six patients in both groups) and the treatment success rate was 88.46% (46 patients in both groups) (Table 2).

**Figure 3:**
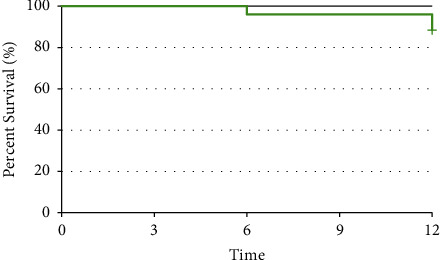
Cumulative survival rate in two groups.

Finally, the data of this study were statistically analyzed by the survival analysis test by the Kaplan–Meier method. This analysis showed that there is no statistically significant difference between the survival rates between the two materials (*P* value = 1, Figure 3).

## 4. Discussion

The results of this study showed that there was no statistically significant difference in terms of clinical and radiographic outcomes between MTA and Biodentine after pulp treatment during a 12-month follow-up.

MTA is a mixture of 3 powdered ingredients of Portland cement (75%), bismuth oxide (20%), and gypsum (5%) [23]. It contains calcium oxide (50–75 wt%) and silicon oxide (15–20 wt%), which together make up 70–95% of cement. Blending of these materials produce tricalcium silicate, tricalcium aluminate, dicalcium silicate, and tetracalcium aluminoferrite [24]. BD is presented in the form of a capsule in which the ratio of powder to liquid is observed. The powder of this substance is composed of tricalcium silicate (3CaO.SiO_2_, main core material), dicalcium silicate (2CaO.SiO_2_, second core material), calcium carbonate (CaCO_2_, filler), zirconium oxide (ZrO_2_, radio-opacifier), and iron oxide (coloring agent). Its liquid contains calcium chloride, which accelerates the function of a water-soluble polymer as a water-reducing agent. The concentration of the constituents of this substance is not provided by the manufacturer [21].

Both BD and MTA induce mineral foci formation and the early odontoblastic differentiation, so they can form reparative dentin synthesis. These two substances also cause the secretion of the growth factor TGF-*β*1 from pulp cells, which probably involved in reparative dentine synthesis [18].

The findings of this study are consistent with previous studies on the effect of MTA and Biodentine on pulp treatments.

A study by Cuadros-Fernández et al. [25] in 2016 showed that both materials had similar clinical results and above 90%, and that among MTA-treated primary teeth, only one tooth in 12-month follow-up underwent internal root resorption and two teeth from the group treated with Biodentine underwent internal root resorption and periapical radiolucency, which was not statistically significant.

In 2017, Juneja and Kulkarni [26] compared the outcomes of new bioactive materials with traditional pulp treatments, such as formocresol, and showed that the clinical and radiographic effects of MTA and Biodentine were similar in pulp treatments for 18 months and both were superior to formocresol, which was a statistically significant difference.

On the other hand, in the study of Carti [27] in 2018, the clinical and radiographic success of the two bioactive materials, MTA and Biodentine, in the treatment of pulpotomy of the primary molars was similar.

The findings of a 2019 study by Çelik et al. [28], which treated the mandibular primary pulp using MTA and Biodentine, showed that in the long-term follow-up of 24 months, the clinical and radiographic results of MTA were 100%, and that of Biodentine is 89.4%. Therefore, similar to the findings of the present study, there was no significant difference between the two materials. As a result, both materials can be used to treat primary molars that take a long time to exfoliate normally.

On the other hand, the results of the clinical study of Uesrichai et al. [29] in 2019, which examined the effects of two bioactive materials PRO ROOT MTA and Biodentine in the treatment of permanent teeth with symptoms and irreversible pulpitis with 36-month follow-up, were very similar to the results of the present study. In this way, Biodentine has no lower clinical and radiographic success than PRO ROOT MTA, which is a standard gold material, and the effects of both materials are similar to each other.

In 2019, a systematic review and meta-analysis of 9 clinical articles showed that MTA and Biodentine have no superiority over each other [30].

In 2020, Abuelniel et al. [31] compared the effect of MTA and Biodentine as pulpotomy materials in the treatment of traumatized immature anterior permanent teeth. They showed that both MTA and Biodentine have similar clinical and radiographic outcomes but discoloration was significantly more in the MTA group.

Various clinical trial studies with long-term follow-up have shown that conservative pulp treatments using new bioactive materials in highly decayed and highly symptomatic teeth had high success and can be a definitive treatment. Obstacles to MTA such as discoloration, difficult application, high material cost, and long setting time, Biodentine application due to less tooth discoloration, easier application, and much shorter setting time can be a very good alternative. Further studies on the use of Biodentine in symptomatic primary teeth are suggested with more samples and longer follow-up. Studies on different teeth are also recommended.

## 5. Conclusions

The results of this and previous studies showed that the properties of Biodentine are similar to MTA, and both materials have suitable sealing properties of pulp, antibacterial, dentine tissue production, and induction of regeneration and proliferation of pulp cells. Both of these bioactive materials showed a high rate of clinical and radiographic success.

## Figures and Tables

**Table 1:** Frequency distribution of outcomes measures at 6 and 12 months in two groups.

Material	MTA (*n* = 26)		Biodentine (*n* = 26)		*P* value
Time	6 months	12 months	6 months	12 months	
Percussion sensitivity	1 (3.85%)	1 (3.85%)	0	1 (3.85%)	1
PDL widening	0	1 (3.85%)	1 (3.85%)	0	1
External root resorption	0	0	0	1 (3.85%)	1
Spontaneous pain	0	0	0	0	1
Abscess (fistula)	0	0	0	0	1
Pathological loosening	0	0	0	0	1
Periapical radiolucency	0	0	0	0	1
Internal root resorption	0	0	0	0	1
Furcal radiolucency	0	0	0	0	1
Lack of integrity of laminate dura	0	0	0	0	1

**Table 2:** Comparison of the frequency distribution of success and failure between the two groups.

Groups	Group 1 (MTA, *n* = 26)	Group 2 (Biodentine, *n* = 26)	Total (*n* = 52)
Frequency of success	23 (88.46%)	23 (88.46%)	46 (88.46%)
Frequency of failure	3 (11.54%)	3 (11.54%)	6 (11.54%)

## Data Availability

The datasets generated and/or analyzed during the current study are available from the corresponding author on reasonable request.
